# Airway Rescue and Extended Recovery in Prone Nonintubated Anesthesia: A Narcolepsy Type 2 Case Report

**DOI:** 10.7759/cureus.115375

**Published:** 2026-08-28

**Authors:** Yueh-Ting Lai, Tsai-Wei Chen, Shih-Chieh Chung

**Affiliations:** 1 Anesthesiology, E-Da Hospital, I-Shou University, Kaohsiung, TWN

**Keywords:** airway rescue, anesthesia, case report, high-flow nasal oxygen, narcolepsy type 2, prone position, remimazolam

## Abstract

Narcolepsy presents unique perioperative challenges, particularly during postoperative recovery, while the prone position limits airway access. We present the case of a 32-year-old woman with untreated narcolepsy who underwent prone jackknife hemorrhoidectomy after declining neuraxial anesthesia. Following preoperative counseling and awake positioning, nonintubated intravenous anesthesia was maintained with remimazolam and propofol during spontaneous ventilation with high-flow nasal oxygen. A stop-and-supine airway rescue plan was prepared. She regained full orientation 13 min following anesthetic discontinuation and reported no worsening of daytime sleepiness or unintended sleep episodes during one-month follow-up. Airway rescue, emergence, and narcolepsy-related recovery were assessed as separate perioperative considerations.

## Introduction

Most perioperative evidence in narcolepsy originates from case reports and rarely distinguishes disease subtype, medication status, or coexisting sleep apnea. Concerns remain regarding unpredictable responses to sedatives and delayed emergence from anesthesia. One systematic review linked favorable outcomes with continuation of established narcolepsy medication, short-acting agents, multimodal analgesia, and depth-of-anesthesia monitoring [1]. However, a matched case-control study reported intraoperative and phase I recovery similar to that in controls, whereas emergency response team activations were more frequent later in the perioperative period [2]. This indicates that perioperative risk may arise after emergence rather than during it; therefore, immediate anesthetic recovery and later narcolepsy-related recovery should not be treated as one outcome. Prone nonintubated anesthesia poses another challenge since airway access is limited after positioning. In this case report, we describe how airway rescue, anesthetic emergence, and postoperative symptom assessment are planned together in a patient with narcolepsy type 2 undergoing prone jackknife hemorrhoidectomy. This report adheres to the CARE reporting guideline.

## Case presentation

A 32-year-old woman (height, 159.5 cm; weight, 47.4 kg; body mass index, 18.6 kg/m²; American Society of Anesthesiologists physical status II), with a clinical diagnosis of narcolepsy type 2, established approximately five years earlier, presented for excisional hemorrhoidectomy. Overnight polysomnography recorded total sleep time of 471 min, with normal apnea-hypopnea and oxygen desaturation indices. Multiple sleep latency testing showed reduced mean sleep latency and four sleep-onset rapid eye movement periods among five naps. Cataplexy was absent, and cerebrospinal fluid orexin had not been measured. There was no family history of narcolepsy or other central disorders of hypersomnolence. She reported sleeping for as long as 16 h per day, although she could be awakened, and experienced fatigue at work. Modafinil and methylphenidate had been discontinued three years earlier owing to chest tightness and nausea and vomiting, respectively. No narcolepsy medication was administered. During the preanesthesia assessment, we discussed possible postoperative worsening of sleepiness or other sleep symptoms and advised her to avoid driving when unusually sleepy or fatigued.

Neuraxial anesthesia was offered and declined. Preoperative airway assessment revealed no predictors of difficult mask ventilation or tracheal intubation. The patient and surgical team agreed on nonintubated intravenous anesthesia with spontaneous ventilation. Since airway access would be limited after positioning, oral and nasopharyngeal airways, a supraglottic airway, and equipment and personnel for tracheal intubation were prepared. Naloxone and flumazenil were immediately available. If oxygenation or ventilation became inadequate, the plan was to stop surgery and the anesthetic infusions, return the patient to the supine position, perform airway maneuvers, and provide positive-pressure ventilation or tracheal intubation if required. Naloxone or flumazenil would be considered if respiratory depression was suspected to be opioid- or remimazolam-related. The patient assumed the prone jackknife position while awake. Proper alignment of the face, elbows, and iliac crests was carefully checked, and arm abduction was limited to prevent stretching the brachial plexus. Finally, patient comfort and the absence of nerve compression symptoms were confirmed prior to anesthetic administration.

Intravenous lidocaine (60 mg) and atropine (0.3 mg) were administered to prepare the patient. Anesthesia was induced with remimazolam (3 mg) and fentanyl (50 µg) and maintained with remimazolam at 15 mg/h. High-flow nasal oxygen was delivered at 60 L/min during spontaneous ventilation. Pulse oximetry, a continuous capnographic waveform, and bispectral index monitoring were also used. Movement during surgical stimulation prompted additional propofol target-controlled infusion using the Schnider model at an effect-site concentration of 1-3 µg/mL, and the bispectral index was maintained at 40-60. Analgesia included intramuscular extended-release dinalbuphine sebacate (150 mg), intravenous propacetamol (2 g), and intravenous parecoxib (40 mg). The surgeon performed perianal infiltration with 2% lidocaine; the volume was not recorded. Palonosetron (0.25 mg) was administered for antiemetic prophylaxis. Total hemorrhoidectomy with wedge resection and skin-tag excision lasted 30 min.

Both anesthetic infusions were terminated at 55 min (Figure 1).

**Figure 1:**
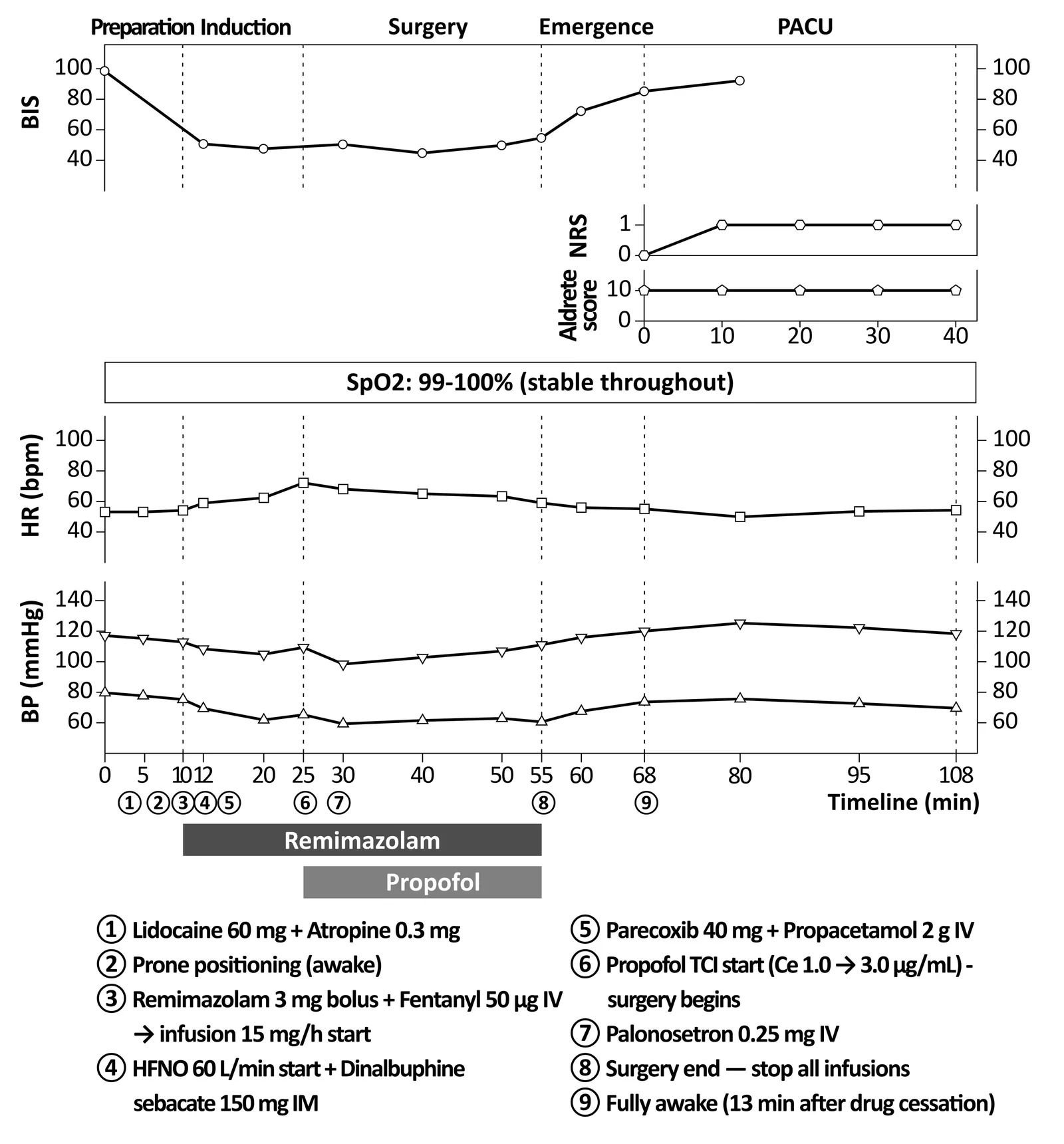
Perioperative anesthetic course and recovery. Bispectral index, heart rate, blood pressure, oxygen saturation, anesthetic infusions, and recovery scores are shown against elapsed time. Both infusions were stopped at 55 min, and the patient was fully awake 13 min later. Pain numerical rating scale scores were 0–1, and Aldrete scores were 10 during postanesthesia care. BIS: bispectral index; BP: blood pressure; Ce: effect-site concentration; HFNO: high-flow nasal oxygen; HR: heart rate; NRS: numerical rating scale; PACU: postanesthesia care unit; TCI: target-controlled infusion.

Thirteen minutes later, without flumazenil, the patient opened her eyes spontaneously, followed verbal commands, and was well oriented in person, place, and time. Oxygen saturation remained at 99%-100%, heart rate at 50-72 beats/min, and blood pressure at 98/59-125/80 mm Hg. No airway maneuver, assisted ventilation, or interruption of surgery was required. During the 40 min in the postanesthesia care unit, her Aldrete score was 10, and her pain score was 0-1 on a 0-10 numerical rating scale. She was admitted for observation with a family member present continuously. No additional rescue opioid was required, and no nausea, increased sleepiness, or respiratory issues were observed. A symptom-directed follow-up was performed by an anesthesiologist during an inpatient visit on postoperative day 1, an outpatient visit on day 7, and by telephone at one month. At each visit, she was asked about daytime sleepiness, unintended sleep episodes, sleep duration, nausea, respiratory symptoms, and return to usual activities. She reported no increase in daytime sleepiness or sleep duration, no unintended sleep episodes, no nausea or respiratory symptoms, and no delay in resuming her usual activities.

## Discussion

The primary take-home message of this case report lies in planning three elements: phenotype-specific assessment and counseling, rescue from an airway event when airway access is limited, and evaluation of later narcolepsy-related symptoms in addition to routine emergence. In this case, a prompt anesthetic recovery and stable narcolepsy-related symptoms through one month were the endpoints we aimed to confirm.

Medication continuation is commonly recommended for patients receiving narcolepsy therapy [1]; however, it did not apply here since both drugs had been terminated years prior due to adverse effects. Defining the phenotype was therefore important. Cataplexy was absent, the sleep studies supported narcolepsy type 2, and the normal apnea-hypopnea index did not suggest coexisting obstructive sleep apnea. A survey of patients with narcolepsy reported limited counseling on possible postoperative sleepiness and drowsy driving [3]. Preoperative counseling and family inpatient observation formed part of the safety plan, while symptom-directed follow-up assessed outcomes that a rapid Aldrete recovery could not capture.

Prone nonintubated anesthesia poses a separate airway challenge. One randomized trial during prone endoscopic sedation found less hypoxemia and fewer procedure interruptions with high-flow nasal oxygen than with a conventional nasal cannula [4]. High-flow oxygen does not secure the airway, and preserved oxygen saturation does not prove adequate ventilation. Due to the lack of reliable numeric end-tidal carbon dioxide values, the capnogram was read only as a qualitative respiratory waveform. Awake self-positioning, immediately available airway devices and intubation capability, and an explicit plan to stop surgery and return the patient supine were central to the technique.

Remimazolam-propofol coadministration has been described in a three-patient case series, in which recovery occurred within 8-13 min [5]. Herein, propofol was added only after movement during stimulation, while remimazolam preserved the option of reversal with flumazenil. A single case, however, cannot show pharmacodynamic synergy or dose reduction. Given the patient's known narcolepsy, analgesia was planned to limit opioid exposure. The non-opioid components included propacetamol and parecoxib, consistent with procedure-specific recommendations for paracetamol plus an NSAID or COX-2 inhibitor [6], and fentanyl was limited to a single dose at induction. Perianal lidocaine infiltration and extended-release dinalbuphine sebacate, a long-acting opioid analgesic whose analgesic activity extends beyond phase I recovery [7], were also used; however, current PROSPECT guidance does not recommend routine perianal local-anesthetic infiltration or intramuscular sebacoyl dinalbuphine ester because of insufficient procedure-specific evidence [6]. The technique should therefore not be described as fully reversible, opioid-free, or reliant only on short-acting drugs. The absence of additional rescue opioids, respiratory events, nausea, or worsening somnolence during inpatient observation and follow-up remains clinically relevant.

Finally, prompt command following and an Aldrete score of 10 indicate early anesthetic recovery, not recovery from narcolepsy-related symptoms. Patient-reported worsening following general anesthesia has been described in central disorders of hypersomnolence [8]; the matched narcolepsy study found adverse events following apparently similar phase I recovery [2]. Our symptom-focused assessments over one month showed no worsening. However, these assessments did not utilize a validated sleepiness scale, and the single observation, along with the brief procedure, makes interpretation challenging. The case does not identify a preferred anesthetic technique.

## Conclusions

In this patient with untreated narcolepsy type 2 undergoing prone nonintubated anesthesia, a prompt anesthetic recovery did not, in itself, confirm recovery from narcolepsy-related symptoms. For comparable patients, we suggest a four-step strategy. First, offer regional or neuraxial anesthesia through shared decision-making; this reduces systemic sedative exposure and is associated with favorable outcomes in narcolepsy. Second, if these techniques are declined or unsuitable, prepare a position-specific airway rescue plan before prone nonintubated anesthesia. Third, counsel the patient preoperatively regarding postoperative sleepiness and drowsy driving. Finally, reassess narcolepsy-related symptoms after discharge, rather than assuming uncomplicated emergence equates to complete recovery.
